# Effects of Farrowing Stall Layout and Number of Heat Lamps on Sow and Piglet Production Performance

**DOI:** 10.3390/ani10020348

**Published:** 2020-02-22

**Authors:** Suzanne M. Leonard, Hongwei Xin, Tami M. Brown-Brandl, Brett C. Ramirez, Somak Dutta, Gary A. Rohrer

**Affiliations:** 1 Department of Agricultural and Biosystems Engineering, Iowa State University, Ames, IA 50011, USA; smleonar@iastate.edu (S.M.L.); bramirez@iastate.edu (B.C.R.); 2 UT AgResearch, University of Tennessee, Knoxville, TN 37996, USA; 3 Department of Biological Systems Engineering, University of Nebraska-Lincoln, Lincoln, NE 68583, USA; 4 Department of Statistics, Iowa State University, Ames, IA 50011, USA; somakd@iastate.edu; 5 USDA, ARS. U.S. Meat Animal Research Center, Clay Center, NE 68933, USA; gary.rohrer@ars.usda.gov

**Keywords:** creep area, daily gain, farrowing design, housing, lactation, litter uniformity, over-lay, pre-weaning mortality, sow crate, stillborn, sow parity

## Abstract

**Simple Summary:**

In the commercial swine industry, farrowing stalls are commonly used as a strategy to reduce piglet pre-weaning mortalities caused by sow over-lay. Farrowing stall dimensions have generally remained the same over the past 50 years in the United States, even though the sizes of both the sows and litters have increased considerably. This extensive field study investigated if sow and piglet productivity would be affected when housed in stalls of traditional layout, additional area for the piglets, or additional area for the sow. All three layouts were also tested with use of one or two supplementary heat lamps in the creep area. Results show that stall layout and number of heat lamps had no statistical impact on production outcomes. However, seasonal differences, sow parity, and number of litter mates did have significant effects. Providing larger stalls or an additional heat lamp costs more for the producer but did not yield production improvements.

**Abstract:**

Most farrowing facilities in the United States use stalls and heat lamps to improve sow and piglet productivity. This study investigated these factors by comparing production outcomes for three different farrowing stall layouts (traditional, expanded creep area, expanded sow area) and use of one or two heat lamps. Data were collected on 427 sows and their litters over one year. Results showed no statistical differences due to experimental treatment for any of the production metrics recorded, excluding percent stillborn. Parity one sows had fewer piglets born alive (*p* < 0.001), lower percent mortality (*p =* 0.001) and over-lay (*p =* 0.003), and a greater number of piglets weaned (*p <* 0.001) with lower average daily weight gain (ADG) (*p <* 0.001) and more uniform litters (*p =* 0.001) as compared to higher parity sows. Farrowing turn, associated with group/seasonal changes, had a significant impact on most of the production metrics measured. Number of piglets born influenced the percent stillborn (*p <* 0.001). Adjusted litter size had a significant impact on percent mortality (*p <* 0.001), percent over-lay (*p <* 0.001), and number of piglets weaned (*p <* 0.001). As the number of piglets weaned per litter increased, both piglet ADG and litter uniformity decreased (*p <* 0.001). This information can be used to guide producers in farrowing facility design.

## 1. Introduction

The commercial US swine industry transitioned to stall farrowing in the 1960s as an effort to reduce pre-weaning piglet mortality [1]. Farrowing in stalls remains the most common indoor system in the US, making this an important area of research [2]. Compared to loose housing systems (pens), farrowing stalls have been shown to lower pre-weaning mortality (PWM) [3]. However, the national average PWM was 17.8% in 2017, demonstrating that the modern swine industry has further opportunities for improvement [4]. High PWM, coupled with increasing sow dimensions, more piglets per litter, and growing public concern over sow welfare in farrowing stalls, indicate that revisiting the space allocation of commercial farrowing environments is warranted [4,5,6]. 

Numerous studies have compared pen to stall farrowing; however, few studies investigated the arrangement, dimensions, and floor area of the piglet creep and sow stall within the farrowing stall [3,7,8]. In conventional farrowing stalls, 1.96 m^2^ of floor area is delineated as piglet creep area, which is within the space recommendations by Wheeler et al. [9]. Limited literature is available on the effects of changing the allotted creep floor area on piglet productivity, but it is well documented that the number of piglets born per litter has steadily increased, thus suggesting that more creep floor area is needed [4,10]. In grow-finish pigs, space allocation has a significant impact on growth performance and it has been shown that piglets farrowed in pens weigh more at weaning than piglets in farrowing stalls [11,12]. This suggests that expanding piglet creep areas in farrowing stalls may provide production benefits. Some farrowing stall manufacturers in the US have begun to offer larger farrowing stalls with an increase of 1.0 m^2^ to the piglet creep area, though there is no scientific data to validate the impact of this additional space. This study provides a scientific evaluation of this increased piglet creep floor area as compared to traditionally sized farrowing stalls.

Information on how dimension and area allocation within a farrowing stall may impact the sow is also lacking. Various measurements of sow body length, width, height, and depth by McGlone et al. suggested that traditional sow stall dimensions may be too narrow to accommodate some commercial sows, in particular, the depth of body when lying laterally for older or late gestation sows [6]. A more recent study in 2011 found similar results, confirming that modern sow dimensions sometimes exceed the provided sow stall dimensions in farrowing stalls [5]. Therefore, providing sow stalls with greater dimensions could potentially improve sow welfare and thus, productivity during farrowing and lactation. In the larger farrowing stalls being produced in the US, sows are provided an additional 0.31 m of stall length compared to traditional farrowing stalls. However, the above literature suggests that it is the width of the stall that may be inadequate. Therefore, the present study investigated the effects of increasing sow stall length as available in commercial configurations, as well as increasing sow stall width. For the increase in width, 0.1 m was added to achieve width recommendations similar to that found in literature [6].

Another important aspect of the farrowing stall environment is supplementary heat sources used to mitigate pre-weaning mortality, which are used to provide a warmer microenvironment to meet the thermal needs of piglets while attracting them away from the sow to reduce incidence of over-laying. Supplemental heat sources can warm piglets, reducing the likelihood of hypothermia and subsequent mortality [13,14]. There are many options and commercially available supplementary heat sources, such as covers, nests, and partially or fully enclosed boxes. However, in the US, the two most common supplementary heat sources are heat lamps and electrically heated mats. Literature indicates that electrically heated mats have greater energy efficiency and subsequently lower operating costs compared to heat lamps, but it has been shown that piglets prefer lamps over electrically heated mats for the first two days after birth [15,16]. Scientific studies comparing the two heat sources found no difference in piglet performance between heat lamps or electrically heat mats [16,17]. 

Heat lamps are ubiquitous in commercial US farrowing systems, as they are easier to manage and cost effective. Therefore, this study opted to provide supplementary heat with heat lamps in order to mimic typical US commercial conditions. Use of two heat lamps per farrowing stall can increase the area of the creep floor that is heated, which in turn increases the likelihood that neonates will be able to locate and benefit from the additional heated area. Placing one heat lamp on either side of the farrowing stall further increases the opportunity newborn piglets have of reaching a heated area. In this manner, regardless of which side of the sow stall piglets travel to first, they could be warmed and dried by a heat lamp. There is limited literature investigating if the additional heated area impacts piglet or sow productivity. Added heat lamps can also potentially lead to increased undesired heating of the sow, which can result in heat stress and reduced milk production [18]. Further work is needed to understand the relationship between the use of an additional heat lamp and sow and piglet productivity.

Considering the continued challenge of pre-weaning piglet mortality and the limited literature investigating the farrowing stall environment, this large-scale field study was conducted. Specifically, this study evaluates the effects of three farrowing stall layouts and use of one or two heat lamps on: (1) PWM, and specifically over-lay, (2) number of piglets born alive and weaned per sow, and (3) average daily weight gain of piglets and litter uniformity. 

## 2. Materials and Methods 

### 2.1. Facilities

Data collection occurred at the United States Department of Agriculture - Agricultural Research Service U.S. Meat Animal Research Center in Clay Center, Nebraska. This site was an integrated farrow to finish swine facility with 1040 sows farrowing per year. Every effort was made to mimic standard operating procedures used in commercial swine production. The site contained two farrowing facilities, one of which was utilized for data collection. 

Each of the farrowing facilities consisted of three rooms, with each room containing 20 individual farrowing stalls aligned in two rows of ten stalls. Rows were arranged such that the heads of the sows were facing each other across a 1.2 m wide alley with additional alleyways behind each row of stalls. The facility was mechanically ventilated with evaporative cooling pads conditioning fresh air entering a common plenum hallway during warm ambient temperatures. Baffles on one endwall distributed fresh air from the hallway into the rooms and air exchange was provided by fans on the opposite endwall. During cold ambient temperatures, supplementary forced air furnaces preheated hallway air and the endwall baffles closed. Fresh air was then delivered by an air plenum suspended from the ceiling that spanned the length of each side of the room. Additional direct-fired combustion forced air furnaces were suspended from the ceiling in each room and were operated as needed. 

The room air temperature set point was 24 °C for the first week of lactation and was gradually lowered to 20 °C by the end of the farrowing cycle. Dry-bulb temperature and relative humidity (RH) were recorded every 10 min at two locations within each room with portable data loggers (XR440, Pace Scientific, Boone, NC, USA) throughout the course of the study. Data loggers were suspended 1.3 m above the floor near the center aisle and were placed between the first two farrowing stalls at the beginning of one row and between the last two stalls at the end of the other row. Three additional loggers located in the interior hallway (one outside each room) recorded inlet air temperature and relative humidity every 10 min. Average room air temperature was 24.1 ± 0.8 °C (mean ± SD) and average RH was 57% ± 21%. Manure was managed through fully slatted metal floors and a sloped shallow pit, separated by room. Each room contained two 1900 L manual dump tanks as part of a fresh water flush system. These tanks were manually flushed twice per day and moved the waste out of the rooms to a waste lagoon.

### 2.2. Management

**Figure 1:**
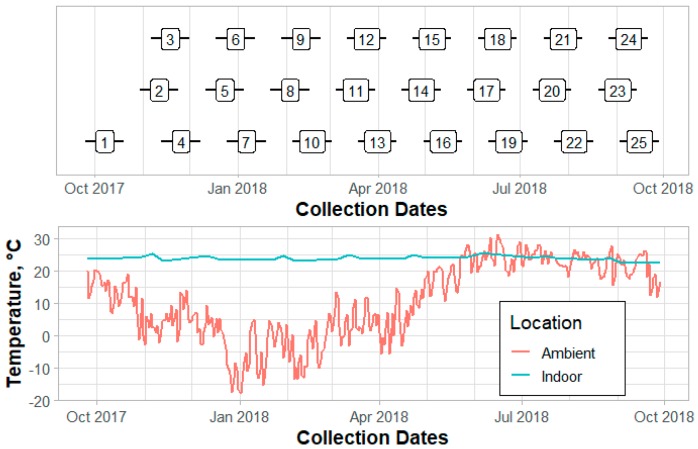
Data were collected on 25 turns of sows entering the farrowing facility. Three farrowing rooms were used and collection occurred from September 2017 to October 2018. Average daily ambient and indoor (average for all three farrowing rooms) air temperatures are displayed.

All animal husbandry protocols were performed in compliance with federal and institutional regulations regarding proper animal care practices and were approved by the U.S. Meat Animal Research Center Institutional Animal Care and Use Committee (2015–2021). Sows were bred to either a commercial Landrace or Yorkshire sire. Data were collected on sows entering the farrowing rooms as gilts or parity (P) 1–3, as all sows were automatically culled after their fourth lactation cycle. All rooms operated on a 6-week cycle per turn, where turn is one farrowing group (all-in, all-out batch) of sows. For each turn, sows were moved into the farrowing room as a group five days prior to anticipated farrowing date and randomly assigned to a stall. Average piglet age at weaning was 26.7 ± 1.9 d. All animals for a given turn were removed from the room as a group and the farrowing room remained empty for nine days for pressure washing and disinfection. The three farrowing rooms used in this study operated on a schedule offset of one week. Data were collected for one year (September 2017 to October 2018) on 25 turns (farrowing groups) of sows, with temporal distribution shown in Figure 1.

Trained animal caretakers followed typical commercial husbandry practices. Specifically, drying mineral powder was sprinkled on mats under supplementary heat lamps. Piglets were weighed and ear tagged on day one. Three days after birth, piglets were tail docked, needle teeth clipped, castrated, and administered iron shots. Cross-fostering of piglets occurred as needed within 3 d after parturition for uniformity of litter sizes. Sows were fed a corn-soybean meal diet once a day until three days after parturition, then *ad libitum*. Creep feed was provided for piglets from 21 days of age until weaning. Drinking water was always available for both sows and piglets via nipple drinkers.

### 2.3. Stall Layouts

Three farrowing stall layouts were tested: traditional (T), expanded creep area (C), and expanded sow and creep areas (S) in combination with either one or two supplementary heat lamps (HL), for a total of six treatments. Farrowing stalls had outer dimensions of 1.83 (W) × 2.5 m (L) and identical metal panels were added to modify the interior dimensions as needed. All sow stalls were 1.2 m tall. The entire floor area was slatted metal, with a slat width of 8.5 mm and a 9.5 mm void space between slats (TriDEK, Hog Slat, Inc.; Newton Grove, NC, USA). The slats featured no-slip grip indentions and 127 × 9.5 mm deep indentions in the sow area to increase traction. This flooring also allowed the sow stalls to be affixed at any position within the farrowing stall to achieve the desired layout. All sow stalls were partitioned from the piglet creep area with horizontal bars. The bottom bar bowed out to allow easier piglet access to the udder and was adjustable in height. Sows stalls also featured anti-crush bars. 

**Figure 2:**
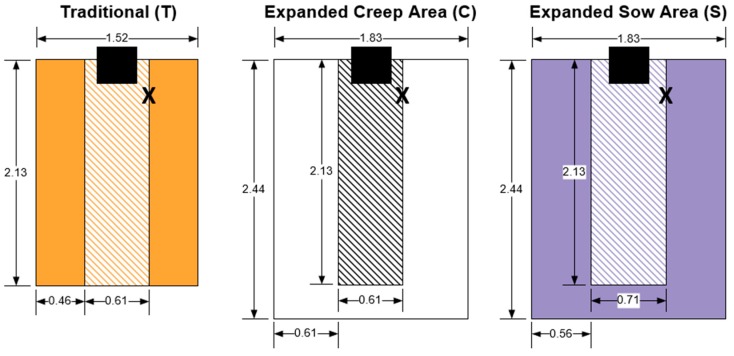
Three experimental farrowing stall layouts used for traditional stall layout (T), expanded creep area layout (C), and expanded sow area layout (S). Shaded areas indicate piglet creep areas and striped areas are sow areas. Sow feeders, shown in solid black, had outer dimensions of (L×W) 0.35 × 0.34 m, of which 0.23 m of feeder length extended into the sow stall area. The “X” symbol represents double water assemblies which were mounted on the bars of the side of the sow stall approximately 0.3 m from the front of the stall. All dimensions are in meters.

For T layout, sows were provided 0.61 × 2.13 m within the outer stall dimensions of 1.52 × 2.13 m. In the C layout, the sow stall remained the same with dimensions of 0.61 × 2.13 m; however, the piglets were provided additional creep area within the outer farrowing stall dimensions of 1.83 × 2.44 m. The third layout, S, had a sow stall of 0.71 × 2.13 m while maintaining outer farrowing stall dimensions of 1.83 × 2.44 m. Stainless steel bowl feeders with outer dimensions of (L×W×H) 34.9 × 34.3 × 38.8 cm were provided in the front of the sow stalls (Farrowing Sow Small Bowl Feeder, Hog Slat, Inc.; Newton Grove, NC, USA). Feeders were mounted such that 23.2 cm of length extended into the sow area. Double water assemblies mounted on the bars of the side of the sow stall approximately 0.3 m from the front of the stall provided nipple drinkers for both sows and piglets. Stall layouts are displayed in Figure 2. 

The T layout was based on recommended farrowing stall design from the Midwest Plan Service Swine Housing and Equipment Handbook, with 1.30 m^2^ floor area for sows and 1.95 m^2^ of floor area for piglets [19]. The C layout provided the same floor area for the sow but had 3.17 m^2^ of piglet creep area (an increase of 1.22 m^2^ compared to T layout). In the S layout, there was greater floor area for both the sow and piglets compared to the T layout. The S layout had 1.51 m^2^ sow area and 2.96 m^2^ piglet creep area (an increase of 0.21 m^2^ sow area and 1.01 m^2^ piglet creep area compared to T layout). The S layout provided 0.21 m^2^ greater sow area and reduced piglet creep area by 0.21 m^2^ as compared to the C layout. Note that in each stall layout, 0.08 m^2^ of the sow floor area was occupied by the sow feeder. 

**Figure 3:**
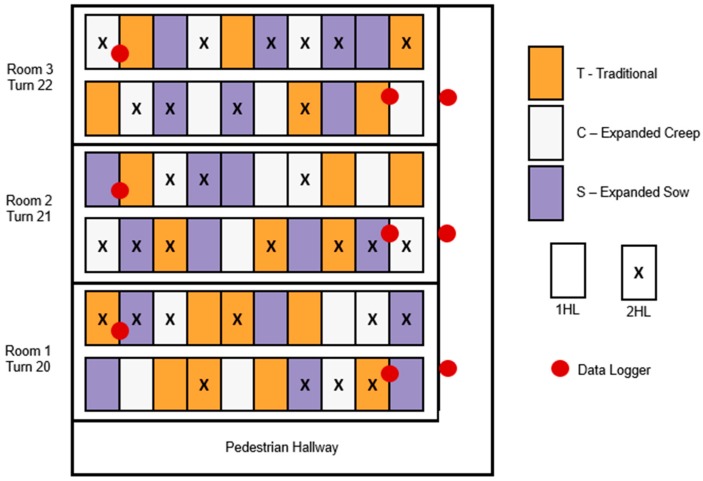
Experimental layout for arbitrarily selected turns 20-22. Stall layouts were randomized once and remained constant throughout the study, while heat lamp treatments were re-randomized for each turn.

Farrowing stall layout treatments were randomized among the three rooms. Due to the labor input required to arrange them, the stall layout configuration within all three farrowing rooms remained constant for the duration of the experiment. All available stalls were utilized for data collection; thus, not all layout treatments were present in equal numbers in each room. Layout treatments were evenly distributed by position within row among all rooms. Figure 3 shows the layout treatment assignments by position within the farrowing facility. 

### 2.4. Heat Lamps

Supplementary heat for piglets was provided with a 175 W infrared heat lamp (HL). Two heat source treatments were tested: one HL per stall (1HL treatment) and two HLs per stall (2HL treatment). Each HL was covered by a metal shroud and suspended 0.53 m above the creep area floor, directly above a 0.30 × 1.22 m black rubber mat. Evaluation of thermal images determined the HLs provided an adequate microenvironment for the piglets [20]. For the 1HL treatment, the HL was centered front to back and left to right in the creep area along the side of the sow stall. For the 2HL treatment, one HL was suspended on both sides of the sow stall such that the creep areas on both sides were heated. The HL treatment assignments were randomized for each turn and balanced to represent each treatment as evenly as possible within each turn, and to avoid creating localized hot spots (i.e., putting all 2HL treatments on the same side of the room) (Figure 3).

Solid metal partitions between the stalls prevented radiation from HLs from reaching adjacent stalls. Partitions were 0.61 m tall, which was higher than HL mounting height, and were the same length as the stall to isolate the creep area thermal environment. Pre-experiment tests were conducted to ensure HLs did not impact adjacent farrowing stalls. Unoccupied farrowing stalls were equipped with HLs and rubber mats for testing. For one row of stalls, in every other stall the HLs were turned on for 3 h. Then, thermal cameras and handheld infrared thermometers were used to investigate stall conditions that did not have HLs on. These were compared to another row of stalls where no HLs were on to confirm that HLs were not impacting partition walls or adjoining farrowing stalls.

Both HL(s) and rubber mats were placed in the farrowing stalls according to experimental design within two days after the sows were moved into the room. The HLs began operating approximately two days prior to anticipated farrowing date and remained operational until the piglets were approximately 21 days old. All HLs functioned on a room thermostat and were automatically turned off if room air temperature exceeded 5.5 °C above room set point temperature. 

### 2.5. Data Collection

Trained caretakers recorded all key productivity information. Piglets were individually weighed within 24 h after birth and one day prior to weaning. Number, date, and cause of any mortalities that occurred during the study were recorded. Caretakers used specific criteria to determine cause of death of piglets. Stillborn piglets were identified as: found near the back of the sow, pale in color, and typically moderately or completely covered with afterbirth or membrane. Stillborns were classified as having closed mouths with mucus still in the oral cavity and had wet umbilical cords. Piglets that were over-laid were identified as having more color than stillborns, dry bodies with an absence of membrane, and a dry umbilical cord. Over-lay piglets often had a tongue protruding from the mouth and were found in the floor area around the udder, shoulder, or head of the sow. Indicators of piglets that died shortly after birth were bodies located anywhere in the farrowing stall, tongue protruding, and partially dried. Piglets that were identified as mortalities shortly after birth often weighed less than 1 kg. Any medical treatments of both sows and piglets were recorded.

### 2.6. Data Analysis

All data analysis was performed using R statistical software with lmerTest and emmeans packages [21,22,23]. Preliminary data were used to determine number of litters needed to achieve 85% statistical power. This analysis showed that to detect a 3% difference in PWM (one additional piglet weaned per ~3 litters), data for 269 piglets were needed per treatment. With an anticipated weaned litter size of 10 piglets, this indicated that a minimum of 27 litters per treatment were required. It was possible to collect data on 10 litters per treatment every 8 weeks due to the configuration of the facility; thus, a minimum of 30 weeks of data collection were required. However, considering the potential for missing or outlier data and potential seasonal effects, data were collected for 52 weeks. Refer to Figure 1 for temporal distribution of data collection. 

The data for percent stillborn, PWM, and over-lay were not normally distributed; as such, these factors were transformed prior to analysis. The percentages were converted to a proportion value. Numerous litters did not have stillborns, mortalities, or over-lays, so the smallest non-zero value for a given parameter was divided by two and replaced all zero values for that parameter. Then, a logit transform was performed as shown in Equation (1).

Logit = log(proportion × (1 − proportion) ^−1^) (1)

A first order linear model was developed to fit the data to investigate each of the sow and piglet productivity outcomes (i.e., percent stillborn, number live at birth, percent PWM, percent over-lay, number weaned, ADG, and litter uniformity). All models contained factors for stall layout treatment, HL treatment, and the interaction between the stall layout and HL treatments. Sow parity was included as a factor in all models, as well as sow health status as a binary factor of had/did not have recorded health issues or interventions while the sow was in the farrowing facility. Seasonal effects and genetic differences over time were accounted for by including turn number as a factor (i.e., unique number for each group of sows brought into a farrowing room on the same date). Random effects were specified for sow, sire, and stall location within facility. 

Number of piglets farrowed was included as a covariate for models evaluating percent stillborn and number live at birth. Models for percent PWM and number of piglets weaned contained adjusted litter size (number of piglets born alive ± number of cross-fostered piglets) as a covariate. Number of piglets weaned was then incorporated as a covariate into models to analyze piglet ADG and litter uniformity (coefficient of variation; CoV). Individual piglet ADG values were calculated using weight at birth and weaning, and dates of farrowing and weaning. Then, individual piglet ADG values were averaged over the litter to obtain ADG per piglet for analysis. Visualizations of results were generated using the ggplot2 package [24]. 

## 3. Results

### 3.1. Data Description 

Three sow mortalities occurred due to causes not associated with experimental treatments (e.g., lameness, over gorging) and were excluded from the study. Twelve sows weaning no piglets, due to insufficient lactation or all piglets being cross-fostered off or mortalities, were excluded as well (number of sows from each treatment: T, 1HL = 4; T, 2HL = 2; C, 1HL = 2; C, 2HL = 0; S, 1HL = 2; S, 2HL = 2). Two sows were removed for weaning less than five piglets (T, 2HL = 1; S, 2HL = 1) and one sow for having more than 50% stillborn (S, 2HL). Some farrowing turns also had fewer sows due to conception rates or health reasons. The number of usable replicates were balanced by treatment for each turn. 

Overall, a minimum of 68 usable replicates were collected per treatment. Of the 427 total replicates, 409 different sows were observed. This was because 18 sows were subject to data collection twice as they cycled through the production system (at P1 and P3, or P2 and P4). There were no significant differences in sow parity distribution between treatments. On average, the parity distribution was: 41% P1, 26% P2, 16% P3, and 17% P4.

Data on 5895 piglets were collected. Of these, 195 were mummies and 338 stillborn. Piglets were excluded from the analysis due to differing genetic line (27) and incomplete records (14). Due to cross-fostering, at least one piglet was removed from 90 litters and at least one piglet was added to 63 litters. A total of 283 of the 5366 piglets considered in this study were cross-fostered. For ADG and litter uniformity analyses, cross-fosters and any piglets that did not survive to weaning were excluded, leaving 4265 piglets from 427 litters in the dataset.

### 3.2. Results by Treatment

This study aimed to investigate the effects of farrowing stall layout (T, traditional stall layout, C, expanded creep area, or S, expanded sow area) and number of HLs (1HL or 2HL) on sow and piglet productivity. A summary of all production outcomes by treatment can be seen in Table 1. 

All three farrowing stall layout treatments (*p* > 0.15 for all production parameters), HL treatments (*p* > 0.10), and their interaction (*p* > 0.40) were determined to have no statistically significant effect on any of the outcomes measured, except for percent stillborn. Only significant results are reported below. 

### 3.3. Percent Stillborn

**Figure 4:**
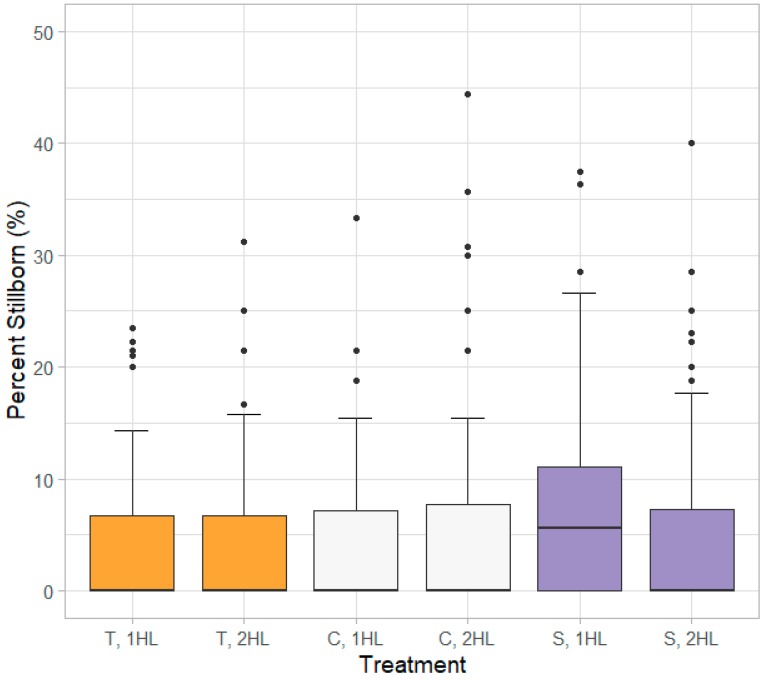
Distribution of percent stillborn piglets by treatment. On the boxplots dark lines within the boxes represent the median value while the box shows the interquartile range. The endpoints of the whiskers show 1.5 times the interquartile range, and dots mark any values that are outside of the whisker range. Stall layouts: traditional (T), expanded creep area (C), expanded sow area (S); Heat lamp treatments: one heat lamp (1HL), two heat lamps (2HL).

The percent of stillborn piglets was significantly affected by farrowing stall layout (*p =* 0.045) (Figure 4). Specifically, piglets in S layouts had 7% increased odds of being stillborn compared to piglets in T layouts. There was evidence that the greater the number of piglets born the greater the percentage of stillborn piglets (*p* < 0.001) and that turn (*p =* 0.06) and sow health status (*p =* 0.07) may have influences as well.

### 3.4. Number Live at Birth

**Figure 5:**
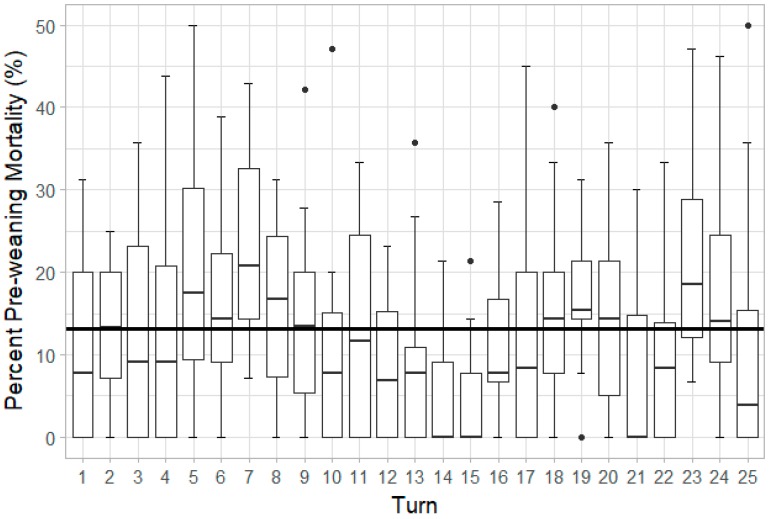
Summary of piglet percent PWM by turn, showing significant seasonal variation (*p <* 0.001). Overall average mortality was 13.1%, shown by the solid black line.

Sow parity had a strong significant influence on the number of piglets live at birth (*p <* 0.001) (Figure 5). In particular, P1 sows farrowed fewer live piglets than P2 (*p =* 0.007), P3 (*p =* 0.003), and P4 sows (*p =* 0.017). No statistical difference was found among other parity comparisons.

### 3.5. Percent Pre-weaning Mortality

Percent PWM was significantly influenced by adjusted litter size (*p <* 0.001), turn (*p <* 0.001), and sow parity (*p <* 0.001). The more piglets that were assigned to a sow to nurse, the greater the percent PWM. Variations in percent PWM by turn are displayed in Figure 5, showing that there were differences due to seasonality. Investigation into parity effects revealed that P4 sows have significantly greater percent PWM than P1 (*p =* 0.001) and P2 (*p =* 0.002) sows.

#### Percent Over-Lay

**Figure 6:**
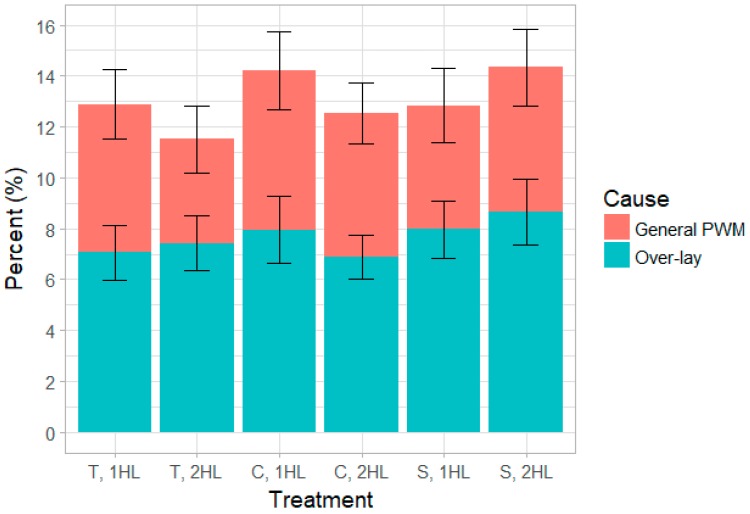
Piglet mortality by cause (general PWM or over-lay) as it occurred within each treatment group. Error bars display standard error by group and mortality cause. Stall layouts: traditional (T), expanded creep area (C), expanded sow area (S); Heat lamp treatments: one heat lamp (1HL), two heat lamps (2HL).

When focusing specifically on PWM attributed to over-lay, there were similar trends to those seen with overall PWM. Mortalities attributed to over-lay accounted for 58% of PWM (Figure 6). Larger adjusted litter size led to greater percent over-lay (*p <* 0.001). Similar trends were seen from turn effects with percent over-lay (*p =* 0.03), as were noted in percent PWM. Sow parity influenced percent over-lay as well (*p =* 0.006), as P4 sows had greater percent over-lay than P1 sows (*p =* 0.003).

### 3.6. Number of Piglets Weaned

**Figure 7:**
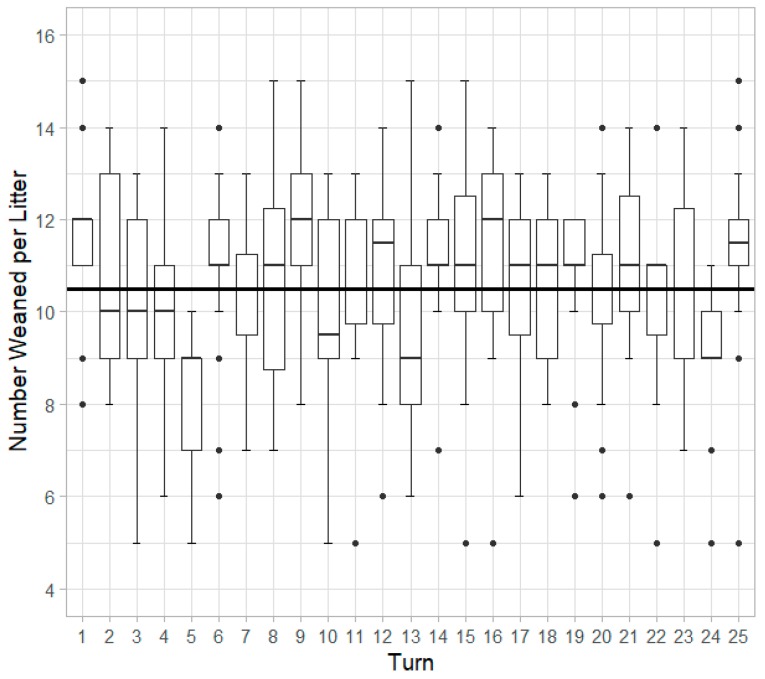
Number of piglets weaned per litter by turn. Overall average number of piglets weaned per litter was 10.5, shown by the solid black line.

Increasing adjusted litter size resulted in a greater number of piglets that were weaned from the litter (*p* < 0.001). Number of piglets weaned was significantly affected by turn (*p =* 0.007) (Figure 7) and parity (*p* < 0.001). P4 sows weaned fewer piglets than both P1 (*p* < 0.001) and P2 sows (*p =* 0.002).

### 3.7. Average Daily Weight Gain (ADG)

The statistical analysis results showed that the more piglets weaned in a litter, the lower the average piglet ADG (*p* < 0.001). Parity also had a significant impact on ADG (*p* < 0.001), with P1 sows producing piglets with lower ADG than P2 (*p* < 0.001), P3 (*p =* 0.04), and P4 sows (*p* < 0.001). There is weak evidence suggesting that P2 sows wean heavier piglets than P4 (*p* = 0.05) (Table 2).

### 3.8. Litter Uniformity

**Figure 8:**
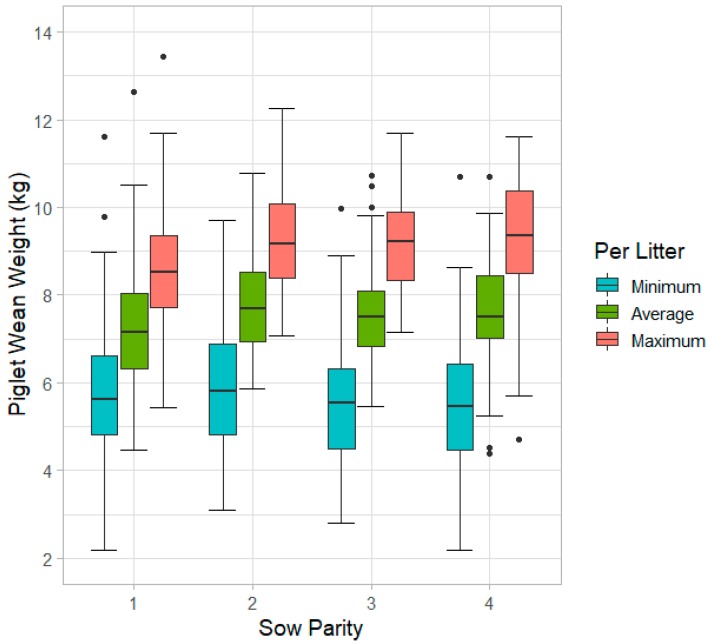
Differences in litter uniformity based on sow parity. Minimum, average, and maximum wean weight of individual piglets within litter are shown.

To investigate the uniformity of litters at weaning, the coefficient of variation was calculated. It was found that litter uniformity decreased with increasing number of piglets weaned (*p* < 0.001). Parity also contributed to litter uniformity (*p* = 0.001). Litters raised by P1 sows had greater uniformity than P4 sows (*p* = 0.001) and tended to be more uniform than P3 sows (*p* = 0.089). There was a trend for P2 litters to have greater uniformity than P4 (*p* = 0.062). Differences in minimum, average, and maximum per piglet weaning weights for each litter are distributed by parity and shown in Figure 8.

## 4. Discussion

There were no meaningful differences observed between traditional farrowing stall layouts and stalls with expanded creep or sow areas. This may not be the case for more prolific animals, as in this study an average of 10.5 piglets were weaned per litter. However, the 2017 industry average was 10.3 piglets weaned per litter, indicating that the information presented here is valid for current commercial conditions [4]. Though no statistical changes in productivity were found, farrowing stall layout and number of HLs could potentially have had an impact on sow and piglet behavior [18,25,26]. This is the focus of a future publication.

Although no statistical differences were found between stall layouts presented in this study, allocating a greater amount of floor area to piglets or sows may be needed to impact production. Baxter et al. suggested minimum sow space allowance of 4.9 m^2^ was needed to meet the biological needs of sows during pre-farrowing, farrowing, and lactation while recommendations of creep area ranged from 0.97–2.32 m^2^ [27]. Another recent publication based on European Union regulations and biological basis recommended a minimum of 5.6 m^2^ floor area for farrowing stalls, of which 1.1 m^2^ minimum should be creep area [28]. These recommendations may indicate that greater areas of space are needed than those provided in the present study to produce significant changes in production or sow and piglet welfare. Though no increase in PWM was noted due to the wider stalls in this study, increases in PWM are seen when further space is provided to sows in pens [3]. Additional scientific study is needed to understand space allocation and its influences on sow and piglet production and welfare. In addition to the quantity of space provided in farrowing stalls, the quality and usability of the space should be considered as well. Flooring and stall partition types can influence the welfare of the sows and piglets by promoting or impeding the expression of natural behaviors [27]. Quality of housing conditions can influence hormone and steroid concentrations in sows as well [12]. Design and delineation of spaces for different activities, such as feeding and dunging, should be based on scientific data in order to best meet the needs of the sows, piglets, and caretakers.

There was no interaction between farrowing stall layout and number of HLs, and no production differences were observed between treatments with one or two HLs. This indicates that using two HLs does not provide any production benefits, making the additional HL a potentially unnecessary extra expense. While this study investigated placing the second HL on the opposite side of the sow stall to heat the creep areas on both sides in an effort to warm piglets regardless of stall side choice, there could be advantages to using a second HL in a different configuration. It is common in industry to place an additional HL near the back of the farrowing stall for the first few days after farrowing. Other anecdotal evidence has suggested that it is possible to increase number of piglets weaned by placing two HLs on the same side of the creep area. Piglets tend to pile together, so providing a larger contiguous heated area may better accommodate this behavior. Future work can further investigate HL placement, and piglet behaviors, to better match the environment to animal preferences to improve performance. 

### 4.1. Percent Stillborn

As mortalities shortly after birth and stillborns are often difficult to discriminate, and farrowings outside typical working hours (06:00 to 15:30) were unattended, it is possible that in some cases piglet cause of death was misclassified [29]. However, as this study was conducted at a research facility, caretakers received in-depth training to reduce misclassifications. Additionally, it is likely that any misclassifications were evenly distributed between treatments.

The statistical difference noted between S and T stall layouts indicates a 7.0% increase in odds of being stillborn when using S stall layouts compared to T stall layouts. On average, the percent stillborn in T stall layouts was 5.1%, so even with the statistical increase in percent stillborn, this is not a meaningful physical difference. The significance of turn effects observed with percent stillborn were similar with the other production outcomes measured in this study, indicating that seasonal and personnel factors have important roles in sow and piglet productivity. Heat stress in warmer summer months can reduce conception rates, resulting in fewer piglets being born during fall months. Personnel factors, such as labor availability and caretaker proficiency and experience levels, can all impact frequency and rigor of daily sow and piglet monitoring. The dynamic influence that these factors can have on piglet productivity is evident based on the results of this study. The number of stillborn piglets increased with increasing number of piglets born in this study, which agrees with other literature [30].

### 4.2. Number Live at Birth

The P1 sows had fewer piglets live at birth compared to P2, P3, and P4 sows, with no statistical differences observed in comparisons amongst the other parities. This indicates that gilts have fewer piglets born alive than experienced sows (Figure 5). This trend is similarly reported in another study on Yorkshire and Landrace sows that found number of piglets live at birth increased until P5 then decreased as parity increased further [31]. Similar results were reported in a larger study of approximately 39,000 sows [32]. Tantasuparuk et al. also found that number of piglets live at birth was significantly less for P1 compared to P2 to P7, further validating the trend found in the present study [33]. 

### 4.3. Percent Pre-weaning Mortality 

The calculated PWM and over-lay percentage were determined using the adjusted litter size, defined as the number of piglets born live and any additions or subtractions due to cross-fostering. Average PWM in this investigation was 13.1%, which is less than the US industry average of 17.8% [4]. This could have an impact on how the study information translates to commercial farms; however, it is still reasonable to provide useful information for commercial producers to make evidence-based infrastructure and management decisions. 

A trend of increasing adjusted litter size and increasing percent PWM was noted. However, cross-fostering was not performed for 65% of the litters in this study. For litters that were not impacted by cross-fostering, this means that the number of piglets born was the same as the adjusted litter size. It may be that number of piglets born is the significant factor, but since the exact timing of mortalities (i.e., before or after cross-fostering occurred) was unknown, adjusted litter size was used in the models to capture the number of piglets assigned to a particular sow stall. A potential explanation for the relationship between adjusted litter size and PWM is that the fewer piglets a sow is nursing, the fewer opportunities for accidental over-lay. Additionally, lower adjusted litter size could result in reduced competition and greater food availability for each piglet. 

Individual turn averages of PWM ranged from 4.3% to 22.7%. The greatest PWM rates were observed in turns 5, 7, and 8 (December 2017 to January 2018), which can be attributed to a disease outbreak, while lower mortality rates in turns 13, 14, and 15 (April to May 2018) likely reflect ideal barn conditions and overall high herd health. A study by Li et al. indicated temperature differences as a potential cause of fluctuations in mortality rates in pen farrowing featuring a mechanically ventilated facility [34]. The study by Li et al. also stated the greatest piglet mortality occurred during warmer ambient conditions when elevated indoor temperatures exceeded the desired thermoneutral conditions [34]. However, the mechanically ventilated facility used in the present study had evaporative cooling pads and produced similar indoor conditions throughout the duration of the experiment (Figure 1). Other factors, such as potential changes in the gestation barn environment, personnel, or feed and water quality, could have contributed as well. Further investigation is needed to identify specific causes. 

Investigation into parity effects revealed that P4 sows have significantly greater percent PWM than P1 and P2 sows. This result is supported by other literature [35]. 

#### Percent Over-Lay

In this study, deaths attributed to over-lay accounted for 58% of piglet PWM (Figure 6). This percentage of overall PWM attributed to over-lay is consistent with findings reported by other studies [30,36]. As expected, larger adjusted litter size produced greater percent over-lay. When a sow is assigned piglets to nurse, there are increased opportunities for potential over-lay. Similar trends were seen from turn effects and sow parity as were noted in percent PWM. P4 sows had greater percent over-lay when compared to P1 sows. These results are consistent with findings from the overall PWM data for this study.

### 4.4. Number of Piglets Weaned

Though larger litters had increased percent PWM and over-lay, the results here indicate that larger adjusted litter size led to a greater number of piglets weaned, showing that larger litter sizes still result in a net increase in prolificacy at the end of lactation. Seasonal effects were determined to have trends inverse to those seen in PWM, which is expected. As PWM increases, it is logical that number of piglets weaned will decrease. P4 sows weaned fewer piglets than both P1 and P2 sows, agreeing with the earlier conclusions that P4 sows have greater PWM rates. 

### 4.5. Average Daily Weight Gain (ADG)

As the experimental facility similarly followed commercial practices, cross-fostering of piglets occasionally occurred within the first three days after parturition. Piglets that were cross-fostered were included in the count of number of piglets born live, adjusted litter size, and number of piglets weaned, but they were excluded from the ADG and litter uniformity analyses. These exclusions were made as cross-fostered piglets incorporated early-life factors that could have had implications on their weight gain, such as different colostrum quality or experimental treatment in their original birth stall than their litter mates.

A decrease in the number of piglets weaned resulted in an increase in individual piglet ADG. Specifically, a piglet that is in a litter of five will gain an additional 0.05 kg day ^−1^ than a piglet in a litter of 10, and an additional 0.10 kg day ^−1^ than a piglet in a litter of 15. Over a 21-day lactation period, this results in cumulative weight gain differences of 1.1 kg and 2.1 kg respectively. Other studies also found that piglets in larger litters had lower individual weight gain [37]. Illman et al. reported that in larger litters there was increased inter-piglet biting and pushing before nursing and more piglets missed the milk ejection event [37]. These findings confirm that larger litters can lead to increased competition and lower milk intake, factors that both contribute to reduced weight gain. As the number of piglets increase it is logical that piglet teat fighting will also increase due to greater competition. Teat fighting has been shown to lead to an increase in sow terminated nursing bouts, confirming that behavioral factors are associated with milk access and thus ADG as well [38]. Cross-fostering and genetic selection can be implemented to increase or decrease average litter size, based on the specific desired production outcomes (more piglets or heavier piglets).

The ADG for litters with P1 sows was lower compared to P2, P3, and P4 sows. This could be a result of younger sows weaning a greater number of piglets, as presented above. However, it could also be an indicator that mature sows are better at lactation than first time gilts. Higher parity sows tend to have higher levels of immunoglobulin G in their colostrum [39]. This means that the piglets of higher parity sows are receiving colostrum of increased quality. Multiparous sows consume more feed which would support a greater volume of milk production, and as a result weaned heavier piglets than primiparous sows [12]. The progeny from gilts have lower weights at birth, weaning, and market compared to the progeny of sows [40]. This indicates that the lighter piglets from gilts weigh less at birth and cannot match the growth rate of sow progeny even at later stages of production. The weak evidence that suggests P2 sows wean heavier piglets than P4 sows observed in this study is likely an artifact of the variance in piglet ADG values from P2 sows.

### 4.6. Litter Uniformity 

Litter uniformity can be an indicator of competition among piglets. Uniformly sized piglets are desirable because there are no large piglets dominating food sources or smaller piglets that are undernourished and not growing on schedule. Having similarly sized piglets at weaning can make mixing less stressful in the nursery stage of production as there will be less of a competitive edge for larger piglets. 

It was found that as the number of piglets weaned increased, litter uniformity decreased, which agrees with results presented in the literature [41,42]. As litter size increases, there are more piglets to compete for food resources, so it is reasonable to expect greater variance among piglets at the end of lactation. Milligan et al. concluded that this competition with weight differences is indeed a challenge for smaller piglets, and these effects are even more pronounced in large litters [41]. 

Parity also played a role in litter uniformity, as P1 sows had greater uniformity than P3 and P4. This difference in litter uniformity was mainly attributed to increased weight of the average and heaviest piglet in litters of higher parity sows as minimum piglet weight was similar for all parties. The difference between lowest and highest individual piglet weight for P1 sows was (mean ± SE) 2.8 ± 0.09 kg, while this difference was 3.8 ± 0.16 kg for P4 sows. Lower parity sows were reported to have more uniform litters than higher parity sows in other studies as well [35,42]. It is important to note that while lower parity sows may produce litters with greater uniformity, as reported above they also have piglets that weigh less.

## 5. Conclusions

Three farrowing stall layouts (traditional, expanded piglet creep area, and expanded sow area) were tested in conjunction with the use of one or two heat lamps (one heat lamp placed in the creep area on either side of the sow stall). Production data were collected by trained farm staff continuously from September 2017 to October 2018 on 427 sows and their litters.

- Farrowing stalls with expanded creep or expanded sow areas did not yield practical or statistical differences in sow and piglet productivity compared to a traditional farrowing stall layout.
- There were no statistically significant differences in sow and piglet productivity when using one or two heat lamps in the farrowing stall layouts investigated.
- Turn, accounting for group of sows and seasonal effects, was a significant factor for most of the sow and piglet productivity metrics measured. This highlights the importance of long-term experimental studies on the farrowing environment.
- Parity 1 sows demonstrated lower percent PWM and weaned more, lighter piglets than higher parity sows.

From a production standpoint, this study suggests that there is no production benefit from expanded creep (additional 1.22 m^2^ creep floor area) or expand sow (additional 1.01 m^2^ creep, 0.21 m^2^ sow floor area) stalls as compared to traditional farrowing stalls. There was also no statistical production benefit from using two heat lamps compared to one. However, further work is needed to determine if even greater additional space provision would produce a difference in production outcomes. Future studies aim to investigate the potential behavior and welfare impacts of the farrowing stall designs presented in this study. The information provided in the present study can guide producers when designing and evaluating farrowing facilities. It can also be used when developing guidelines for sow and piglet space requirements. 

## Figures and Tables

**Table 1:** Summary of production parameters by treatment (average ± SE). The only statistical difference noted was in percent stillborn between T and S stall layouts (*p* = 0.045, averaged over number of heat lamps). PWM: Pre-weaning mortality. Stall layouts: traditional (T), expanded creep area (C), expanded sow area (S); Heat lamp treatments: one heat lamp (1HL), two heat lamps (2HL).

Production Parameter	Treatment					
	T, 1HL	T, 2HL	C, 1HL	C, 2HL	S, 1HL	S, 2HL
Number of Replicates	69	73	68	76	73	68
Percent Stillborn	4.02 ± 0.82	3.79 ± 0.78	4.65 ± 0.84	5.54 ± 1.02	7.20 ± 1.11	5.49 ± 1.04
Number Live at Birth	12.01 ± 0.45	12.66 ± 0.40	12.35 ± 0.40	12.24 ± 0.42	11.85 ± 0.43	12.29 ± 0.52
Percent PWM ^a^	12.88 ± 1.38	11.11 ± 1.28	14.21 ± 1.54	12.53 ± 1.21	12.84 ± 1.44	14.17 ± 1.50
Percent Over-lay ^b^	7.06 ± 1.06	6.98 ± 0.98	7.95 ± 1.33	6.88 ± 0.87	7.96 ± 1.13	8.62 ± 1.32
Number Weaned	10.54 ± 0.25	10.90 ± 0.23	10.50 ± 0.28	10.55 ± 0.23	10.27 ± 0.25	10.38 ± 0.29
ADG (kg d ^−1^ hd ^−1^)	0.22 ± 0.00	0.22 ± 0.00	0.22 ± 0.00	0.23 ± 0.00	0.23 ± 0.00	0.23 ± 0.00
Litter Uniformity—CoV	14.66 ± 0.79	14.53 ± 0.68	14.50 ± 0.62	13.64 ± 0.60	13.56 ± 0.59	13.97 ± 0.70

^a^ Calculated using number of mortalities divided by number of piglets live a birth ± cross-fostered piglets. ^b^ Calculated using number of over-lays divided by number of piglets live a birth ± cross-fostered piglets.

**Table 2:** Average values of production parameters that were significantly influenced by parity. Average values are presented ±SE and averaged over stall layout, HL treatment, turn, litter size, and sow health status.

Production Parameter	Parity			
	1	2	3	4
**Number Live at Birth**	11.18 ± 3.15 ^a^	12.86 ± 3.67 ^b^	13.28 ± 3.98 ^b^	12.76 ± 4.05 ^b^
**PWM (%)**	9.80 ± 10.56 ^a^	12.63 ± 11.70 ^a^	15.78 ± 11.50	18.24 ± 12.12 ^b^
**Over-lay (%)**	5.10 ± 7.86 ^a^	8.02 ± 9.70	8.98 ± 9.60	11.36 ± 10.45 ^b^
**Number of Piglets Weaned**	10.32 ± 2.24 ^a^	10.99 ± 1.99 ^a^	10.65 ± 2.23	10.15 ± 1.97 ^b^
**ADG (kg d ^−1^ hd ^−1^)**	0.22 ± 0.03 ^a^	0.23 ± 0.03 ^b^	0.22 ± 0.03 ^b^	0.23 ± 0.03 ^b^

^a,b^ Indicate statistically different mean values between parities (*p <* 0.05).
